# Reciprocal epigenetic remodeling controls testicular cancer hypersensitivity to hypomethylating agents and chemotherapy

**DOI:** 10.1002/1878-0261.13096

**Published:** 2021-09-15

**Authors:** Ratnakar Singh, Zeeshan Fazal, Emmanuel Bikorimana, Raya I. Boyd, Cliff Yerby, Megan Tomlin, Hannah Baldwin, Doha Shokry, Andrea K. Corbet, Khadeeja Shahid, Aleyah Hattab, Sarah J. Freemantle, Michael J. Spinella

**Affiliations:** ^1^ Department of Comparative Biosciences University of Illinois at Urbana‐Champaign IL USA; ^2^ Carle Illinois College of Medicine and Cancer Center of Illinois University of Illinois at Urbana‐Champaign IL USA

**Keywords:** 5‐aza deoxycytidine, cisplatin, DNA methylation, epigenetics, H3K27me3, polycomb repressive complex

## Abstract

Testicular germ cell tumors (TGCTs) are aggressive but sensitive to cisplatin‐based chemotherapy. Alternative therapies are needed for tumors refractory to cisplatin with hypomethylating agents providing one possibility. The mechanisms of cisplatin hypersensitivity and resistance in TGCTs remain poorly understood. Recently, it has been shown that TGCTs, even those resistant to cisplatin, are hypersensitive to very low doses of hypomethylating agents including 5‐aza deoxy‐cytosine (5‐aza) and guadecitabine. We undertook a pharmacogenomic approach in order to better understand mechanisms of TGCT hypomethylating agent hypersensitivity by generating a panel of acquired 5‐aza‐resistant TGCT cells and contrasting these to previously generated acquired isogenic cisplatin‐resistant cells from the same parent. Interestingly, there was a reciprocal relationship between cisplatin and 5‐aza sensitivity, with cisplatin resistance associated with increased sensitivity to 5‐aza and 5‐aza resistance associated with increased sensitivity to cisplatin. Unbiased transcriptome analysis revealed 5‐aza‐resistant cells strongly downregulated polycomb target gene expression, the exact opposite of the finding for cisplatin‐resistant cells, which upregulated polycomb target genes. This was associated with a dramatic increase in H3K27me3 and decrease in DNMT3B levels in 5‐aza‐resistant cells, the exact opposite changes seen in cisplatin‐resistant cells. Evidence is presented that reciprocal regulation of polycomb and DNMT3B may be initiated by changes in DNMT3B levels as DNMT3B knockdown alone in parental cells resulted in increased expression of H3K27me3, EZH2, and BMI1, conferred 5‐aza resistance and cisplatin sensitization, and mediated genome‐wide repression of polycomb target gene expression. Finally, genome‐wide analysis revealed that 5‐aza‐resistant, cisplatin‐resistant, and DNMT3B‐knockdown cells alter the expression of a common set of polycomb target genes. This study highlights that reciprocal epigenetic changes mediated by DNMT3B and polycomb may be a key driver of the unique cisplatin and 5‐aza hypersensitivity of TGCTs and suggests that distinct epigenetic vulnerabilities may exist for pharmacological targeting of TGCTs.

## Graphical abstract

Alternative therapies are needed for testicular germ cell tumors (TGCTs) refractory to cisplatin. Recent preclinical and clinical studies suggest that cisplatin refractory TGCTs are distinctly sensitive to hypomethylating agents. The current study suggests a reciprocal relationship between cisplatin and hypomethylation agent sensitivity in TGCTs involving DNMT3B and the polycomb pathway that could lead to biomarkers for future clinical trials.

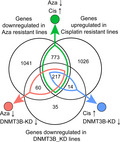

**Abbreviations**
5‐aza: 5‐aza deoxy‐cytosine
BART: binding analysis for regulation of transcription
EC: embryonal carcinoma
FDR: false discovery rate
GSEA: gene set enrichment analysis
TGCTs: testicular germ cell tumors

## 1 Introduction

Testicular germ cell tumors (TGCTs) are the most common solid tumor of males 15–40 years of age [1]. Prior to the introduction of cisplatin‐based chemotherapy, the majority of metastatic TGCT patients died of progressive disease. This situation has been remarkably reversed with cisplatin‐based therapy where nearly 80% of metastatic patients survive beyond 5 years [2]. A greater understanding of the curability of TGCTs may inform more effective strategies to treat other metastatic cancers. Also, cisplatin refractory patients have a poor prognosis and 50% will die of progressive disease [3]. There are essentially no effective traditional, targeted, or immunotherapies for these patients. We and others have shown in preclinical models that TGCT cells are hypersensitive to hypomethylating agents including 5‐aza deoxycytidine (5‐aza) and the 5‐aza prodrug, guadecitabine [4, 5, 6, 7, 8]. This hypersensitivity is on the order of 100‐ to 1000‐fold compared to the sensitivity of other solid cancers, extends to cisplatin‐resistant TGCT cells, and depends on very high levels of the DNA methyltransferase, DNMT3B. These findings are in line with the hypothesis that due to their germ cell origins and low mutational burden, TGCTs may be especially driven and sustained by distinct epigenetic alterations resulting in distinct vulnerabilities to epigenetic drugs [9, 10, 11]. Importantly, hypomethylating agents have been shown to resensitize cisplatin‐resistant cells to cisplatin and we and a second group have provided early phase clinical findings suggesting that a subset of cisplatin refractory TGCT patients favorably respond to guadecitabine in combination with cisplatin [4, 5, 6, 7, 12, 13, 14]. However, besides the potential role of DNMT3B, the mechanisms accounting for the hypersensitivity of TGCT cells to hypomethylating agents are unknown.

In prior work, we developed a series of isogenic cisplatin refractory cell lines from parental cisplatin sensitive cells following a protocol designed to mimic cisplatin‐based patient therapy [15]. Genome‐wide transcriptome analysis revealed that the majority of cisplatin‐resistant cells gained expression of genes normally repressed by the polycomb pathway, which coincided with a global decrease in H3K27me3, H2AUbK119, and decreased expression of the polycomb pathway components EZH2 and/or BMI1 [15]. Further, pharmacologic inhibition of EZH2 conferred cisplatin resistance to TGCT cells, while inhibition of H3K27me3 demethylases sensitized TGCT cells to cisplatin [15]. This implies that targeting the polycomb pathway may have therapeutic value in treating cisplatin‐resistant TGCTs. We also reported that these cisplatin‐resistant cells have global and genome‐wide DNA hypermethylation compared to parental, cisplatin‐sensitive cells [16]. The association between genome‐wide DNA hypermethylation and cisplatin resistance has also recently been demonstrated in TGCT patients [17]. Our cisplatin‐resistant cells also possessed hypomethylation at polycomb target gene promoters, suggesting a complex crosstalk exists between the polycomb and DNA methylation pathways that influences cisplatin sensitivity of TGCT cells [16].

The current study directly addresses mechanisms of TGCT hypersensitivity to DNA hypomethylating agents through the derivation of unique isogenic 5‐aza‐resistant cells models. Interestingly, transcriptome analysis strongly suggests that the polycomb pathway is also altered in 5‐aza‐resistant cells but in a manner opposite to that of isogenic cisplatin‐resistant cells, whereby 5‐aza resistance is associated with a dramatic downregulation of polycomb target gene expression. This reciprocal relationship was also evident for H3K27me3 and DNMT3B with 5‐aza resistance associated with a dramatic downregulation of DNMT3B and upregulation of H3K27me3, the exact opposite situation noted in isogenic cisplatin‐resistant cells. Further, 5‐aza‐resistant cells were more sensitive to cisplatin, while cisplatin‐resistant cells were more sensitive to 5‐aza. Finally, we provide evidence that DNMT3B may be an important upstream driver of epigenetic crosstalk in TGCT cells since DNMT3B depletion alone upregulated the polycomb pathway and conferred resistance to 5‐aza and increased sensitivity to cisplatin. These data suggest that epigenetic remodeling mediated by polycomb and DNMT3B are major drivers of the unique pharmacology of TGCTs that includes hypersensitivity to cisplatin and hypomethylating agents.

## 2 Materials and methods

### 2.1 Derivation of 5‐aza resistant cells

**Fig. 1:**
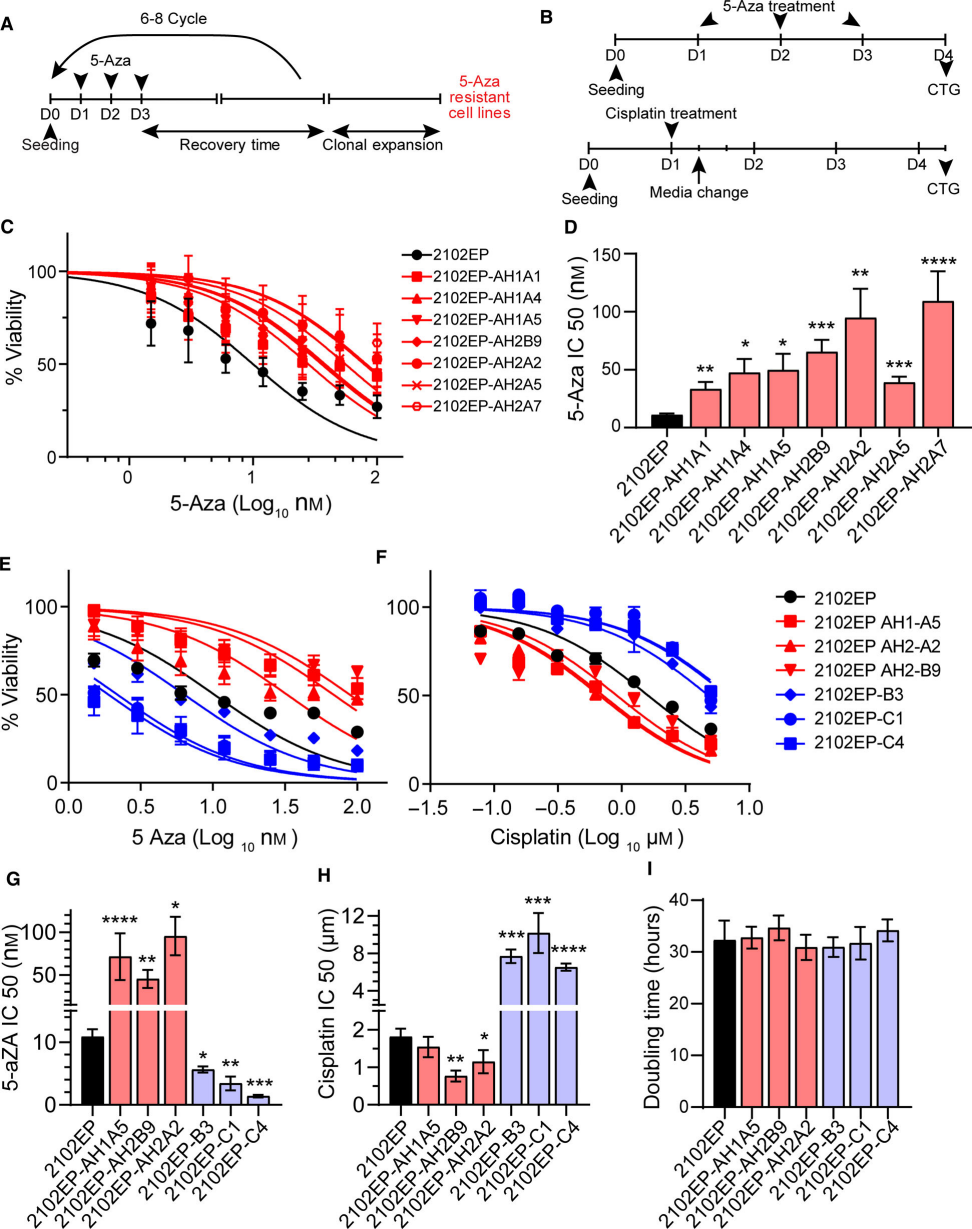
Derivation of 5‐aza‐resistant testicular cancer cells reveals reciprocal drug sensitivities between 5‐aza‐ and cisplatin‐resistant cells. (A) Human testicular cancer derived EC cell line 2102EP was exposed to stepwise dosages of 5‐aza starting at 1 nm and then allowed to recover for 1–2 weeks between doses. After selection in 100 nm 5‐aza, cells were cloned. (B) Schematic of 5‐aza and cisplatin treatment protocols for cell viability assays. CTG, CellTiter‐Glo. (C) 5‐aza‐selected cell lines are stably resistant to 5‐aza. Cell survival and viability were measured using CellTiter‐Glo in parental or acquired 5‐aza‐resistant TGCT cells treated with indicated 5‐aza doses for 3 days. (D) 5‐aza IC50 values for parental and 5‐aza‐resistant cells were estimated from a best‐fit dose–response model. (E) Cisplatin‐resistant cell lines 2102EP‐B3, 2102EP‐C1, and 2102EP‐C4 have increased sensitivity to 5‐aza compared to parental cells. Cell survival and viability were measured using CellTiter‐Glo in parental, 5‐aza‐resistant, and cisplatin‐resistant TGCT cells treated with indicated 5‐aza doses for 3 days. (F) 5‐aza‐resistant cell lines 2102EP‐AH2A5, 2102EPAH2‐B9, and 2102EP‐AH2A2 have increased sensitivity to cisplatin compared to parental cells. Cell survival and viability were measured using CellTiter‐Glo in parental, 5‐aza‐resistant, and cisplatin‐resistant TGCT cells treated with cisplatin for 6 h and assayed 24 h later. (G) 5‐aza IC50 values for parental, 5‐aza‐resistant, and cisplatin‐resistant 2102EP cells. (H) Cisplatin IC50 values for parental, 5‐aza‐resistant, and cisplatin‐resistant 2102EP cells. (I) 5‐aza‐resistant and cisplatin‐resistant 2102EP cells have similar basal doubling times compared to parental cells. All data represent mean ± standard error of the mean of four biological determinations. Two‐tailed Student's *t*‐tests were performed for statistical analysis. *P ≤ 0.05, **P ≤ 0.01, ***P ≤ 0.005, ****P ≤ 0.001 compared to parental cells. All experiments were repeated at least twice with similar results.

All cells were cultured in DMEM (Gibco; Grand Island, NY, USA) with 10% FBS (Invitrogen; Carlsbad, CA, USA). The 2102EP cells are a human testicular cancer‐derived embryonal carcinoma (EC) cell line purchased from ATCC (Manassas, VA, USA) and authenticated by ATCC with karyotyping and short tandem repeat profiling. Cells were frozen within 1 month of purchase and used within 2 months of resuscitation. Generation of 5‐aza resistant cell lines was similar to our previously reported generation of cisplatin‐resistant cells [15]. Parental cells were exposed to stepwise dosages of 5‐aza deoxycytidine (Sigma; St. Louis, MO, USA) starting at 1.0 nm for three consecutive days and then allowed to recover for 1–2 weeks (Fig. 1A). This cycle was repeated 4–6 times with a final selection at 100 nm 5‐aza. Clones were then derived from each pool with cloning cylinders. Cells with prefix AH1, AH2A, and AH2B were cloned from independently treated batches of cells. All clones were stably resistant since resistance to 5‐aza was retained after passaging in 5‐aza‐free media for at least 4 months. Stable cisplatin‐resistant cells 2102EP‐B3, 2102EP‐C1, and 2102EP‐C4 were previously described [15].

### 2.2 Drug treatments and cell viability and proliferation assays

Cells were treated with the indicated dosages of 5‐aza for three consecutive days and cells assayed for survival 1 day after the last dose (Fig. 1B). Cells were treated with cisplatin for 6 h and cells assayed for survival 3 days later (Fig. 1B). To assess cell viability, CellTiter‐Glo (Promega; Madison, WI, USA) assays were performed as previously described [15]. For each cell line, four biological replicates were tested at each concentration, and experiments were repeated at least twice on different days. IC50 values were estimated from the best‐fit dose–response model selected by residual standard error using graphpad prism software (San Diego, CA, USA). To estimate cell doubling times, equal number of cells were plated in 24‐well plates and viable cell numbers determined for four consecutive days using the CellTiter‐Glo assay. Doubling times were calculated with an exponential growth curve equation using graphpad prism software.

### 2.3 Lentiviral shRNA knockdown

Lentiviruses were produced by co‐transfecting HEK293 cells with 10 μg of the viral packaging vector pCMV‐dR8.2 and envelope vector pCMV‐VSV‐G (10 : 1 ratio) and 10 μg of lenti‐shRNA or the control vector using Lipofectamine 3000. Lentiviral silencing shRNA targeting DNMT3B (TRCN0000437183, TRCN0000414235, TRCN0000378379, TRCN0000364153, and TRCN0000364152) was purchased from Sigma along with TRC lentiviral nontargeting shRNA control (SHC016). The HEK293 cell medium was changed 24 h after transfection, and cells were incubated for 48 h to allow for virus production. After 48 h, HEK293 medium containing viral particles was filtered and transferred onto 2102EP cells for 48 h. After transduction, cells were selected with 2 μg·mL^−1^ puromycin.

### 2.4 RNA‐sequencing

RNA was extracted from indicated parental, 5‐aza‐resistant, DNMT3Bsh1‐knockdown, and shPLK0.1 control cells in biological triplicate using the RNeasy Mini Kit (Qiagen; Germantown, MD, USA). RNA sequencing was performed by the Roy J. Carver Biotechnology Center. RNA‐Seq libraries were prepared using the TruSeq Stranded mRNA Sample Prep kit as previously described. The libraries were sequenced on a HiSeq 4000 using HiSeq 4000 sequencing kit version 1. Initial quality control was performed using fastqc. Trimmomatic was used to remove low‐quality bases from both ends LEADING ≤ 28 and TRAILING ≤ 28, respectively, with minimum length of 30. The reads in fastq format were aligned to human genome assembly NCBI GRCh38.p13 using star aligner as previously described [15]. Reads were counted and assigned to genes using featureCount. The limma r package was used to identify differentially expressed genes [18]. Genes whose expression was not greater than 0.5 counts per million in at least two samples were removed, and the resultant filtered expression matrix was TMM‐normalized. Benjamini–Hochberg false discovery rate (FDR) was used to correct for multiple hypotheses. Genes with FDR ≤ 0.01 and absolute fold change ≥ 1.5 were considered differentially expressed. In all cases, each 5‐aza‐resistant cell line was compared to the parental line. The ‘enhanced volcano’ r package was used for visualization of volcano plots. RNA‐seq data for 2102EP‐C1 cells were from our prior study [15]. The RNA‐seq datasets for the current study have been submitted to the NCBI Database of GEO Datasets under the accession numbers GSE172266 and GSE172458.

### 2.5 Downstream enrichment analysis

Gene set enrichment analysis (GSEA) from the Broad Institute was performed to identify enriched gene sets in each resistant cell line compared to parental cells [19]. The BART (Binding Analysis for Regulation of Transcription) tool in gene‐set mode was used to predict the enrichment of transcription factor binding sites in differentially expressed genes [20]. geneoverlap package from r bioconductor was used to identify significant gene set overlap between common gene expression changes between 2102EP‐AH2A2, 2102EP‐C1, and 2102EP‐DNMT3BKD cells and C2 gene sets from the MSigDB database [19, 21].

### 2.6 Western analysis and real‐time PCR

For western analysis, cells were lysed in radioimmune precipitation buffer and separated by SDS/PAGE. Antibodies to actin (MA1‐744; Thermo Fisher; Waltham, MA, USA), ubiquitin H2A‐K119 (3240; Cell Signaling Technology; Danvers, MA, USA), H3K27me3 (9733; Cell Signaling Technology), BMI1 (6964; Cell Signaling Technology), EZH2 (5246; Cell Signaling Technology), DNMT3B (ab2851; Abcam; Cambridge, UK), and DNMT1 (SC‐20701; Santa Cruz Biotechnology; Dallas, TX, USA) were used. Total cellular RNA was isolated using the RNeasy Mini Kit (Qiagen), and complementary DNAs were synthesized using iScript Reverse Transcription Supermix (Bio‐Rad Laboratories; Hercules, CA, USA). Quantitative real‐time PCR assays were performed with iTaq Universal SYBR Green Supermix (Bio‐Rad Laboratories) and the QuantStudio 3 Real‐time System (Thermo Fisher). In all cases, gene expression was normalized to β‐actin. Primers for RT‐PCR are provided in Table S1.

### 2.7 Statistics

Two‐tailed Student's *t*‐tests were performed where appropriate using graphpad prism v6.0, and *P*‐values indicative of nonsignificant *P* > 0.05 and significant *P ≤ 0.05 were determined. Mean and standard error of the mean was used to describe sample variability.

## 3 Results

### 3.1 Derivation of acquired 5‐aza‐resistant testicular cancer cells reveals reciprocal drug sensitivities between 5‐aza‐ and cisplatin‐resistant cells

We and others have previously documented that TGCT‐derived EC cells undergo an acute cytotoxic response to low nanomolar levels of hypomethylating agents including 5‐aza and guadecitabine [4, 5, 6, 7, 8]. Anticancer effects for most somatic cancer cells require doses of hypomethylating agents 100‐ to 1000‐fold higher and the effects at these higher doses manifest as delayed antiproliferative responses rather than acute cytotoxic responses [22]. In order to better understand the mechanism of hypomethylating agent hypersensitivity of EC, we developed a panel of distinct clonal 5‐aza‐resistant cell lines from parental 2102EP cells. 2102EP cells were chosen as it was difficult to generate 5‐aza‐resistant cells in other parental TGCT lines, including NT2/D1 and 833K without inducing differentiation. Cells were treated with increasing dosages of 5‐aza starting at 1 nm for three consecutive days and allowed to recover for 1–2 weeks. Final selection was at 100 nm 5‐aza, and cells were then cloned (Fig. 1A). Cells with prefix designation AH1, AH2A, and AH2B were cloned from independently treated batches of cells. Resistance to 5‐aza was confirmed in dose response experiments with 3 day 5‐aza treatments (Fig. 1B,C). The 5‐aza IC50s of resistant cells ranged from 35 to 100 nm compared to the 10 nm IC50 of parental cells (Fig. 1D). Resistance was maintained for at least 4 months of cell passage in 5‐aza free media.

In a prior report, we noted that one of the most 5‐aza‐sensitive EC cell lines was the cisplatin resistant EC line 833K‐CP [6]. This prompted us to compare and contrast 5‐aza and cisplatin sensitivity in the newly generated 5‐aza‐resistant 2102EP cells with our previously derived isogenic cisplatin‐resistant 2102EP cells [15]. We focused our studies on three 5‐aza‐resistant lines (2102EP‐AH2A2, 21012EP‐AH2B9, and 2102EP‐AH1A5) and three cisplatin‐resistant lines (2102EP‐B3, 2102EP‐C1, and 2102EP‐C4) [15]. All three cisplatin‐resistant cell derivatives demonstrated increased sensitivity to 5‐aza compared to parental 2102EP, while all three 5‐aza‐resistant cells lines demonstrated either increased sensitivity to cisplatin or a trend toward increased sensitivity to cisplatin (Fig. 1E–H). This was despite the fact the all six cell lines had comparable levels of basal cell proliferation when drug was not present as compared to parental cells, indicating that differences in drug sensitivity was not due to gross alterations in cell proliferation (Fig. 1I).

### 3.2 Acquired 5‐aza resistance in testicular cancer cells is associated with genome‐wide alterations in levels of polycomb target genes, H3K27me3, H2AUbK119, and DNMT3B that is in an opposing manner compared to cisplatin resistant cells

**Fig. 2:**
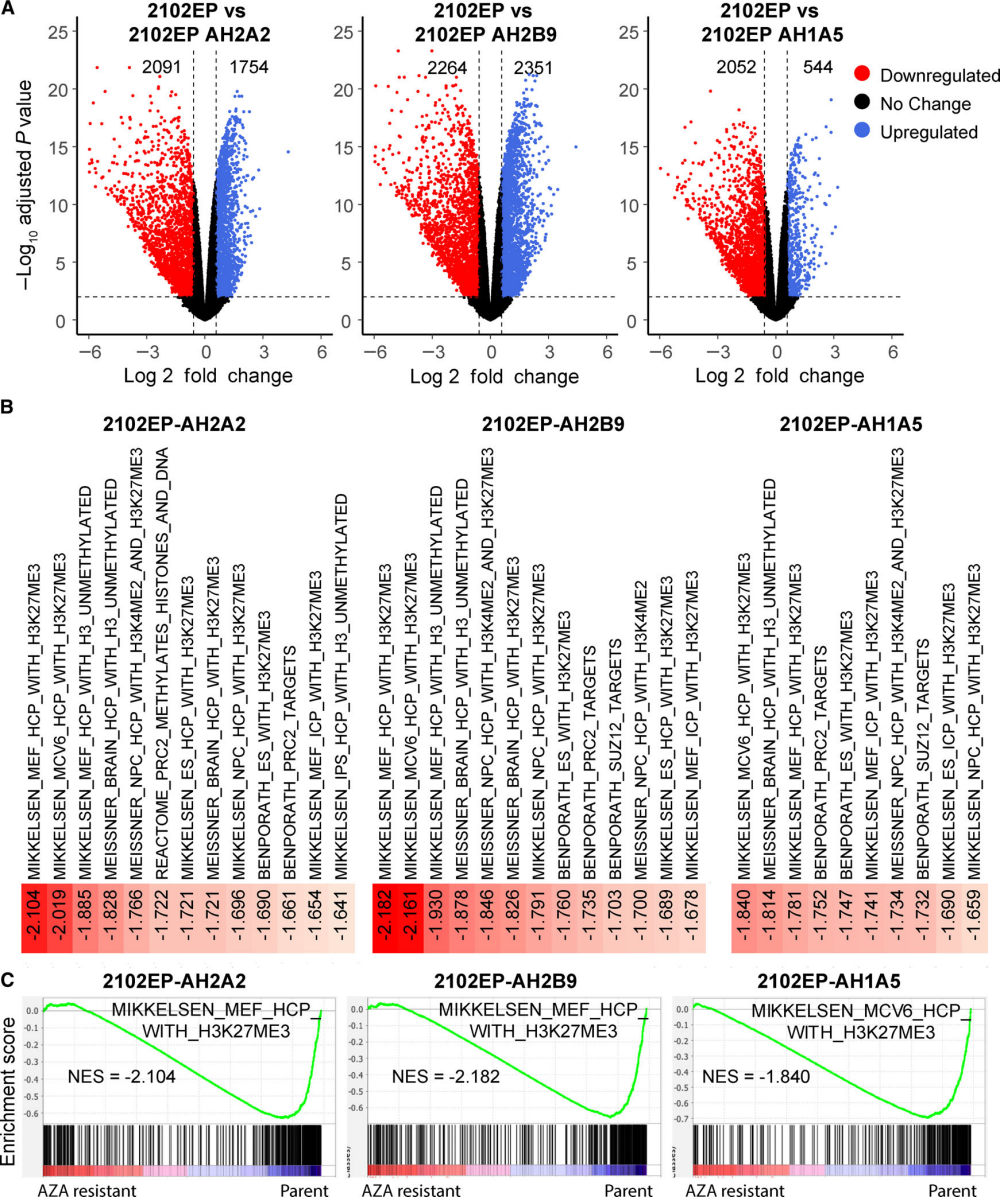
5‐aza resistance in testicular cancer cells is associated with decreased expression of polycomb target genes. (A) Volcano plots indicating the number of significantly upregulated (blue) and downregulated (red) genes in 5‐aza‐resistant 2102EP‐AHA2A, 2102EP‐AH2B9, and 2102EP‐AH1A5 cells compared to parental 2102EP cells. Number of genes upregulated and downregulated with a 1.5‐fold cutoff and FDR ≤ 0.01 for each resistant line is indicated. Volcano plots are based on RNA‐seq analysis from biological triplicates. (B) Downregulated genes from 5‐aza‐resistant cells are enriched for gene sets associated with H3K27 methylation and PRC2 targets. GSEA indicating all polycomb‐related negatively enriched gene sets within the top 50 ranked by NES from 5529 curated gene gets from the MSigDB C2 collection in 5‐aza‐resistant cells compared to respective parental cells. (C) Representative gene set enrichment plots. NES, normalized enrichment score.

In order to understand mechanisms of 5‐aza resistance, RNA‐seq analysis was performed in biological triplicate in 2102EP parental and 2102EP‐AH1A5, 2102EP‐AH2B9, and 2102EP‐AH2A2 cells. There was a substantial number of differentially expressed genes with a fold‐change of 1.5 and FDR ≤ 0.01 in 5‐aza‐resistant cells as compared to parental 2102EP cells (Fig. 2A). For AH1A2 and AH2B9 cells, there were 2091 and 2264 genes downregulated, respectively, compared to parental cells and 1754 and 2351 genes upregulated, respectively. In AH1A5 cells, there was substantially more genes downregulated than upregulated compared to parental cells at this cutoff, 2052 vs 544 genes, respectively. The identity of these differentially expressed genes is provided in Table S2.

We next performed unbiased GSEA comparing each aza‐resistant cell line with parental 2102EP cells. The top 50 positively and negatively enriched gene sets (from over 5529 curated gene sets) for each cell line ranked by normalized enrichment score is provided in Table S3. Interestingly for genes downregulated in the 5‐aza‐resistant cells, there was a highly significant enrichment for multiple gene sets related to polycomb target genes (Fig. 2B). For example, of the top 50 ranked gene sets for downregulated genes, 13, 13, and 10 independent gene sets related to polycomb target genes were noted for AH2A2, AH2B9 and AH1A5 cells, respectively (Fig. 2B,C). This was by far the most frequent and significant gene set family that emerged from GSEA, suggesting that the 5‐aza‐resistant cells have globally downregulated expression of polycomb target genes. Interestingly, we identified previously in 10 isogenic cisplatin‐resistant EC cells including those derived from parental 2102EP, the exact opposite pattern of polycomb gene expression with polycomb target genes being among the most significantly upregulated gene sets [15].

BART (binding analysis for regulation of transcription) predicts functional transcription factor binding based on more than 6000 ChIP‐seq datasets for over 400 factors in human and mouse cells [20]. The BART analysis of genes downregulated in 5‐aza‐resistant cells, as compared with parental controls, strongly predicted polycomb complex component binding. The number of distinct PRC1/PRC2‐related components contained in the top 20 predicted transcription factor binders for each line ranged from three to five and included EZH2, JARID2, SUZ12, and EED (Table S4). These results further suggest the polycomb pathway is a major mediator in regulating downregulated genes in the 5‐aza‐resistant cells. In contrast, BART analysis of cisplatin‐resistant cells demonstrated polycomb component binding enrichment in upregulated genes [15].

**Fig. 3:**
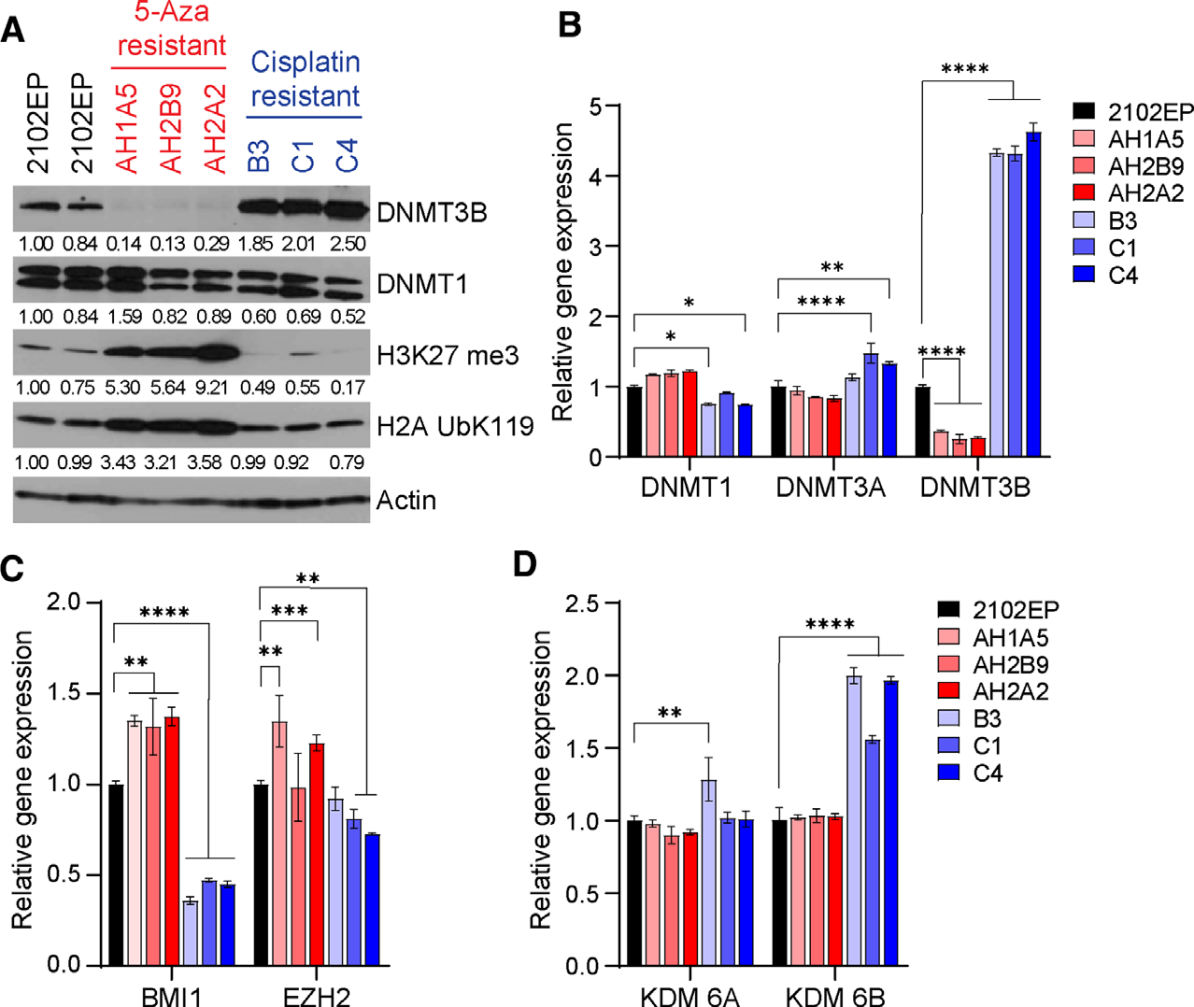
5‐aza‐resistant and cisplatin‐resistant testicular cancer cells have reciprocally altered levels of H3K27me3 and DNMT3B. (A) Immunoblot analysis of indicated cell lines (2102EP parent is loaded twice) with antibodies recognizing DNMT3B, DNMT1, H3K27me3, H2AUbK119, and actin. (B–D) Real‐time PCR analysis of mRNA expression of DNMT1, DNMT3A, DNMT3B, BMI1, EZH2, KDM6A, and KDM6B. Data are the mean of triplicate determinations, and error bars are standard error of the mean. Two‐tailed Student's *t*‐tests were performed for statistical analysis. *P ≤ 0.05, **P ≤ 0.01, ***P ≤ 0.005, ****P ≤ 0.001. Each experiment was repeated at least twice with similar results.

To further probe the reciprocal nature of 5‐aza‐ and cisplatin‐resistant cells, immunoblot and RT‐PCR analyses were performed. We focused on the three 5‐aza‐ and three cisplatin‐resistant 2102EP lines used for current and prior RNA‐seq analysis (Fig. 2A) [15]. Remarkably, all three 5‐aza‐resistant 2102EP cell lines had a dramatic increase in the polycomb marks H3K27me3 and H2AUbK119 compared to parental cells, while, as previously shown, the cisplatin‐resistant cells had decreased expression of these marks (Fig. 3A) [15]. DNMT3B levels were also assessed, since we have previously shown that hypersensitivity of EC cells to 5‐aza is dependent on high‐level expression of DNMT3B [5, 6]. Interestingly, all three 5‐aza‐resistant cells had dramatically decreased expression of DNMT3B, while all three cisplatin‐resistant cells had a dramatic increase in DNMT3B expression compared to parental cells (Fig. 3A). In contrast, there was minimal change in DNMT1 expression among the cell lines.

Real‐time PCR analysis demonstrated substantial downregulated expression of DNMT3B in 5‐aza‐resistant cells and a dramatic upregulated expression of DNMT3B in cisplatin‐resistant cells, consistent with immunoblot analysis and suggesting regulation of DNMT3B in these cell models is at the level of mRNA regulation (Fig. 3B). In contrast, there were only small changes in mRNA for DNMT1 and DNMT3A between the cell lines. PRC1 component BMI1 was upregulated in 5‐aza‐resistant cells while downregulated in cisplatin‐resistant cells consistent with the changes observed in H2AUbK119 (Fig. 3C). Expression of EZH2 also followed this same general trend but was less consistently changed between the resistant cells (Fig. 3C). The H3K27me3 demethylase KDM6B was upregulated in cisplatin‐resistant cells consistent with the downregulation of H3K27me3 in cisplatin‐resistant cells, but unchanged in the 5‐aza‐resistant cell lines (Fig. 3D). In contrast, expression of the H3K27me3 demethylase KDM6A was relatively unchanged between the lines (Fig. 3D). These data suggest that the reciprocal alterations seen in H3K27me3 and H2AUbK119 could be related to changes in expression of certain polycomb components.

### 3.3 DNMT3B knockdown results in induction of H3K27m3, EZH2, and BMI1 expression and confers 5‐aza resistance and cisplatin sensitization to TGCT cells

**Fig. 4:**
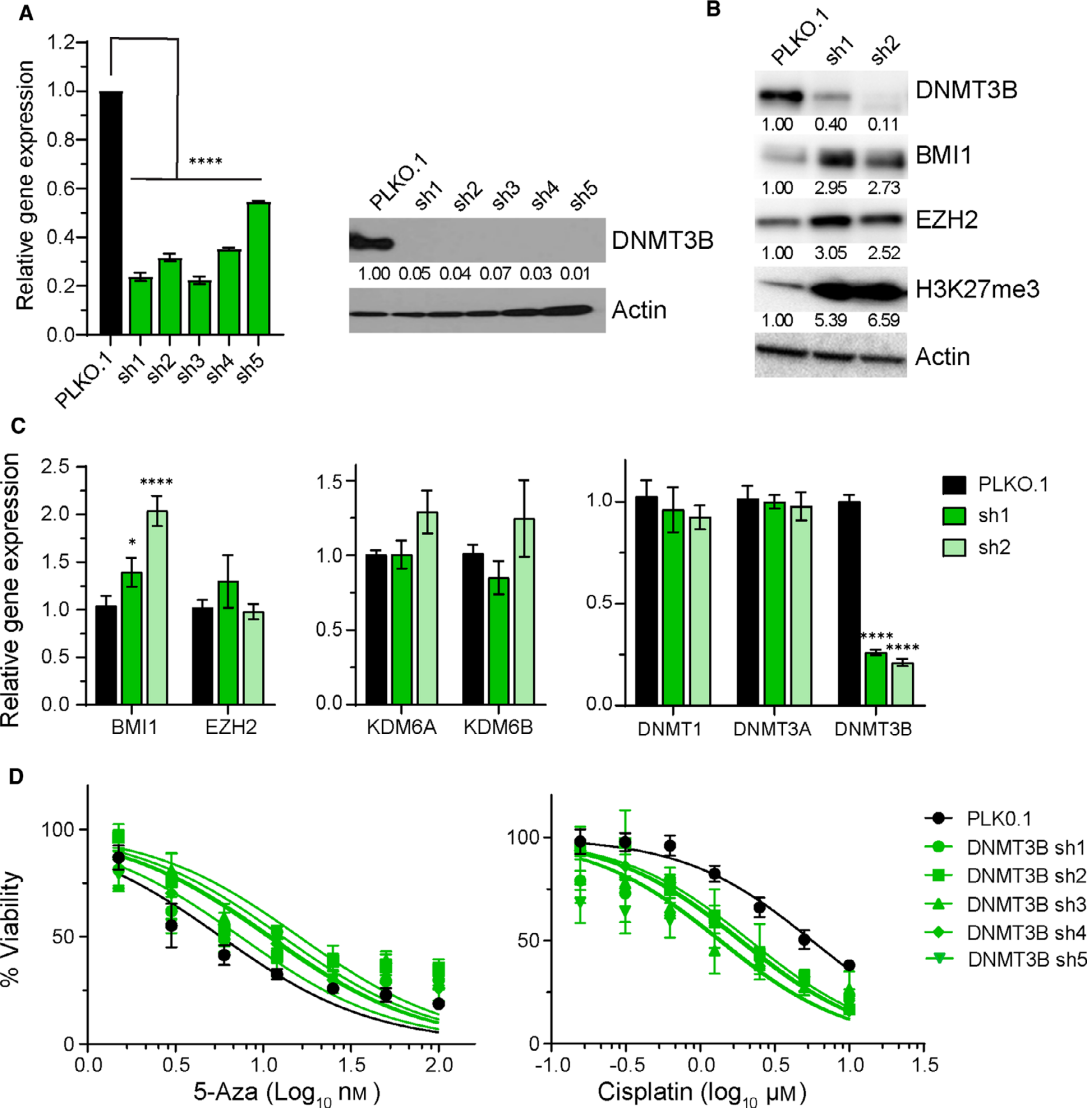
DNMT3B‐knockdown results in induction of H3K27me3, BMI1 and EZH2, and confers 5‐aza resistance and cisplatin sensitization to TGCT cells. (A) Real‐time PCR and immunoblot confirmation of stable shRNA knockdown of DNMT3B in 2102EP cells. (B) Knockdown of DNMT3B in 2102EP cells results in increased protein levels of BMI1, EZH2, and H3K27me3. (C) Real‐time PCR indicates an induction of BMI1 mRNA after DNMT3B knockdown in 2102EP cells. Data are the mean of triplicate determinations, and error bars are standard error of the mean. (D) DNMT3B knockdown in 2102EP cells confers 5‐aza sensitivity and cisplatin sensitization. Data represent mean ± standard error of the mean of four biological determinations. Two‐tailed Student's *t*‐tests were performed for statistical analysis. *P ≤ 0.05, ****P ≤ 0.001. Each experiment was repeated at least twice with similar results.

Our prior work suggested a role for global remodeling of DNA methylation in the upregulation of polycomb target gene expression in cisplatin‐resistant TGCT cells [16]. To investigate whether changes in DNMT3B play a causal role in altering polycomb signaling and 5‐aza and cisplatin sensitivity in TGCT cells, DNMT3B was targeted by shRNA in parental 2102EP cells (Fig. 4A,B). Note that in some experiments shRNA1 only mediated a partial knockdown of DNMT3B. However, DNMT3B knockdown was stable and persisted for extended cell culture intervals up to several months. DNMT3B shRNA resulted in upregulation of H3K27me3 levels and BMI1 and EZH2 protein expression in 2102EP cells (Fig. 4B). BMI1 induction was also noted at the mRNA level where little change was noted in mRNA levels of EZH2, KDM6A, and KDM6B upon DNMT3B knockdown (Fig. 4C). DNMT3B shRNA did not affect levels of DNMT1 and DNMT3A (Fig. 4C). Notably, DNMT3B shRNA also resulted in resistance to 5‐aza, as we have shown previously [5, 6], but importantly these cells also demonstrated a sensitization to cisplatin (Fig. 4D). These results suggest that repression of DNMT3B expression alone is sufficient to induce H3K27me3 levels in TGCT cells and that knockdown of DNMT3B mimics the situation seen in 5‐aza‐resistant cells, namely, 5‐aza resistance and heightened cisplatin sensitivity. The results suggest that alterations in DNMT3B may be driving the crosstalk between DNA methylation and polycomb signaling noted in cisplatin‐resistant and 5‐aza‐resistant TGCT cells.

### 3.4 Substantial genome‐wide overlap between altered polycomb target gene expression in 5‐aza‐resistant, cisplatin‐resistant, and DNMT3B‐knockdown TGCT cells

**Fig. 5:**
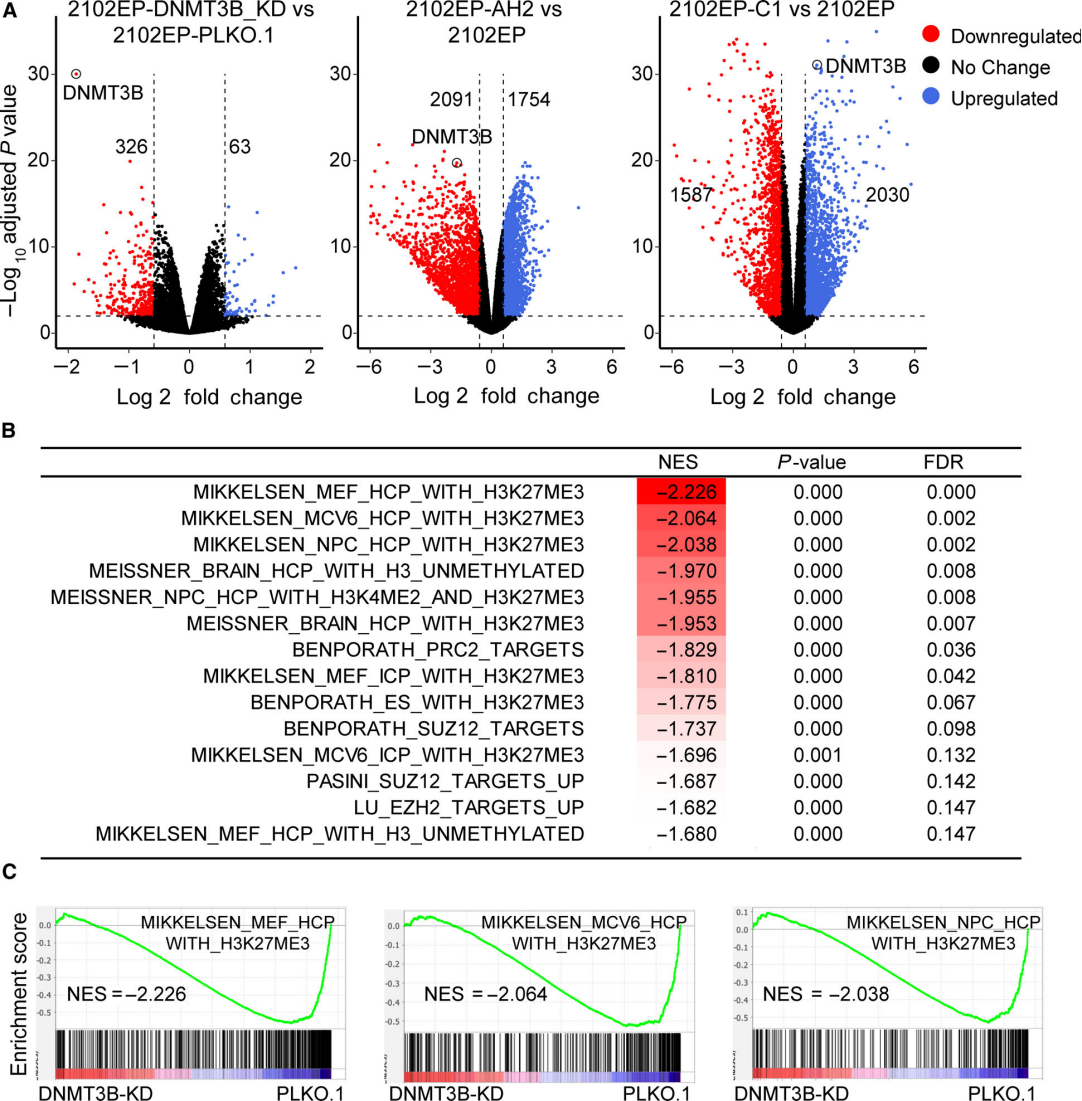
Similar to 5‐aza‐resistant cells, DNMT3B knockdown in TGCT cells results in a genome‐wide decrease in polycomb target gene expression. (A) Volcano plot indicating the number of significantly upregulated (blue) and downregulated (red) genes in 2102EP‐DNMT3B knockdown cells compared to PLK0.1 control cells. Volcano plot of 5‐aza‐resistant 2102EP‐AH2 vs parental and cisplatin‐resistant 2102EP‐C1 cells vs parent is provided for comparison. Number of genes upregulated and downregulated with a 1.5‐fold cutoff and FDR ≤ 0.01 for each resistant line is indicated. Volcano plots are based on RNA‐seq analysis from biological triplicates. The DNMT3B gene is indicated by circle. (B) Downregulated genes from 2102EP‐DNMT3BKD cells are enriched for gene sets associated with H3K27 methylation and polycomb targets. GSEA indicating all polycomb related negatively enriched gene sets within the top 50 ranked by NES from 5529 curated gene gets from the MSigDB C2 collection in 2102EP‐DNMT3BKD cells compared to PLK0.1 control cells. (C) Representative gene set enrichment plots. NES, normalized enrichment score.

Our findings suggest a reciprocal regulation of polycomb signaling in cisplatin‐resistant and 5‐aza‐resistant cells. Since 5‐aza resistance could be phenocopied by DNMT3B silencing alone, RNA‐seq analysis was performed in 2102EP‐DNMT3BKD cells in order to directly compare the transcriptomes of 5‐aza‐resistant, cisplatin‐resistant, and DNMT3B‐knockdown cells. We chose 2102EP‐AH2A2 and 2102EP‐C1 cells as representative 5‐aza and cisplatin cell lines, respectively, for this analysis. Interestingly, at a cutoff of 1.5‐fold and FDR ≤ 0.01, there were many more genes downregulated than upregulated in 2102EP‐DNMT3BKD cells compared to PLK0.1 control cells, 326 downregulated genes, and 63 upregulated genes (Fig. 5A). This is interesting since it conflicts with the canonical role of DNMT3B in facilitating gene silencing by promoter DNA methylation [23]. This trend was present but to a lesser extent in 5‐aza‐resistant 2102EP‐AH2A2 cells with 2091 genes downregulated and 1754 genes upregulated compared to parental cells and reversed to a certain extent in cisplatin‐resistant 2102EP‐C1 cells, with 2030 upregulated genes and 1587 downregulated genes compared to parental cells (Fig. 5A). This suggests that gene expression in 2102EP‐DNMT3BKD cells may be more similar to 2102EP‐AH2A2 cells than to 2102EP‐C1 cells. Differentially expressed genes in 2102EP‐DNMT3BKD cells are provided in Table S5.

Gene set enrichment analysis demonstrated that genes downregulated in 2102EP‐DNMT3BKD cells were highly significantly enriched for multiple polycomb target gene sets, further supporting that DNMT3B‐knockdown mimics global expression changes in 5‐aza‐resistant cells. Of the top 50 ranked gene sets for downregulated genes in 2102EP‐DNMT3BKD cells compared to PLK0.1 control cells, 15 gene sets were related to polycomb target genes (Fig. 5B,C). There were no major trends seen for upregulated genes in 2102EP‐DNMT3BKD cells. The top 50 positively and negatively enriched gene sets for 2102EP‐DNMT3BKD ranked by normalized enrichment score is provided in Table S6. This analysis strongly demonstrates that DNMT3B knockdown alone closely mimics the global downregulation of polycomb target genes seen in 5‐aza cells (Fig. 2B,C). We previously reported the reciprocal pattern for cisplatin‐resistant cells including 2102EP‐C1 where these same polycomb target gene sets were enriched in upregulated genes [15]. BART analysis also predicted polycomb component binding at the promoters of the downregulated genes in 2102EP‐DNMT3BKD cells, which included EZH2, JARID2, and SUZ12 in the top 20 predicted binders (Table S7).

**Fig. 6:**
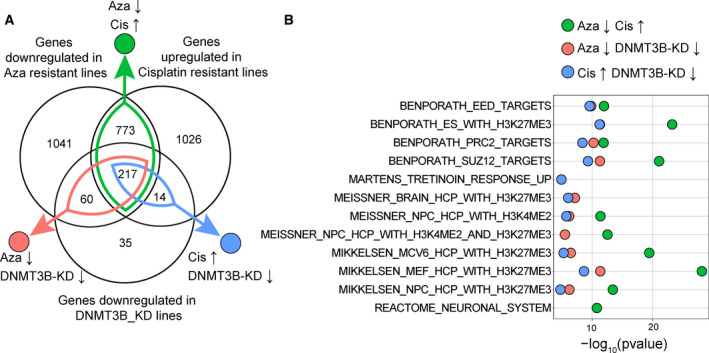
Genome‐wide overlap analysis between 5‐aza‐resistant, cisplatin‐resistant, and DNMT3B knockdown TGCT cells reveals mutual enrichments in the same polycomb target genes. (A) Venn diagram indicating overlap in differentially expressed genes between genes downregulated in 5‐aza‐resistant 2102EP‐AH2A2 cells, upregulated in cisplatin‐resistant 2102EP‐C1 cells, and downregulated in 2102EP‐DNMT3B knockdown cells. Differentially expressed genes are defined as changed 1.5‐fold or greater and an FDR of ≤ 0.01 compared to parental cells or shRNA control cells, respectively. (B) Fisher exact tests against the 5529 curated gene sets of the Broad MSigDB C2 collection performed on the overlap genes between downregulated in 5‐aza‐resistant cells and upregulated in cisplatin‐resistant cells (green), downregulated in 5‐aza‐resistant cells and downregulated in DNMT3B‐KD cells (red), and upregulated in cisplatin‐resistant cells and downregulated in DNMT3B‐KD cells (blue). The top 10 gene sets for each comparison based on *P* value are presented.

Thus far, the data suggest there is a reciprocal relationship in polycomb target genes expression between 5‐aza‐ and cisplatin‐resistant cells with polycomb targets downregulated in 5‐aza‐resistant cells and upregulated in cisplatin‐resistant cells. Furthermore, the data suggest that downregulation of polycomb target genes also occurred in DNMT3B‐knockdown cells. To investigate this relationship further, we analyzed the degree of overlap in differentially expressed genes (1.5‐fold change, FDR ≤ 0.01 compared to parental control cells) between 2102EP‐AH2A2, 2102EP‐C1, and 2102EP‐DNMT3BKD cells. The identity of the overlap genes is provided in Table S8. Approximately 47% of the genes downregulated in 5‐aza‐resistant cells and 49% of the genes upregulated in cisplatin‐resistant cells overlapped between the two cell lines (Fig. 6A). Remarkably, of the genes downregulated in DNMT3B knockdown cells, 85% were also downregulated in 5‐aza‐resistant cells and 75% were also upregulated in cisplatin‐resistant cells (Fig. 6A). These overlap genes were highly enriched in polycomb target genes sets from the GSEA database (Fig. 6B and Table S9). These data suggest that DNMT3B‐knockdown results in downregulation of the same polycomb target genes downregulated in 5‐aza‐resistant cells and upregulated in cisplatin‐resistant cells.

## 4 Discussion

Testicular germ cell tumors can be cured at a high‐rate with conventional cisplatin‐based therapies. However, patients resistant to cisplatin have a very poor prognosis [3]. The mechanisms to account for the sensitivity and resistance of TGCTs to cisplatin are unclear [24, 25, 26]. Although there are no effective alternative or targeted therapies available for cisplatin refractory TGCT patients, recent preclinical and early clinical trial data suggest a subset of cisplatin refractory TGCTs may be hypersensitive to DNA hypomethylating agents [4, 5, 6, 7, 8, 12, 13, 14]. The mechanisms accounting for the hypersensitivity of TGCT cells to hypomethylating agents are entirely unknown apart from a reported dependence on high‐level expression of DNMT3B [6, 7, 8, 9]. Very few studies have addressed the issue of resistance to hypomethylating agents, and these are mainly centered on acute myeloid leukemia [27, 28]. Biomarkers for both cisplatin and hypomethylating agents in TGCT patients are critically needed as early trials have indicated that only a subset of cisplatin refractory patients may benefit from hypomethylating agents [12, 13, 14].

The present study directly addresses mechanisms of TGCT hypersensitivity to DNA hypomethylating agents through the derivation of unique isogenic 5‐aza‐resistant cell models. Transcriptome analysis revealed that 5‐aza resistance is associated with H3K27me3‐mediated downregulation of polycomb target genes and downregulation of the expression of DNMT3B. In addition, *de novo* pharmacogenomic studies in the isogenic 5‐aza‐resistant cells and prior isogenic cisplatin‐resistant cells revealed that the polycomb pathway and DNA methylation are opposing interconnected epigenetic drivers of cisplatin and hypomethylating agent sensitivity in TGCTs. Increased 5‐aza sensitivity in cisplatin‐resistant cells validates prior anecdotal evidence that cisplatin resistance may sensitize TGCT cells to hypomethylating agents and suggests that polycomb/DNA methylation remodeling is one mechanism to account for hypomethylating therapy hypersensitivity in cisplatin refractory TGCTs. Reciprocally and unexpectedly, 5‐aza‐resistant cells were more sensitive to cisplatin. Together, these findings provide further rationale for the combined use of these two therapeutics for treating TGCTs. We have also begun to generate 5‐aza‐resistant cells from other parental TGCT cells, although this has been difficult while avoiding terminal differentiation. Some of these lines also appear to have similar polycomb alterations as in 5‐aza‐resistant 2102EP cells, but to a lesser extent. This suggests that there are likely other mechanisms of 5‐aza resistance in TGCT cells.

We previously showed that H3K27me3 was downregulated with cisplatin resistance and that cisplatin‐resistant cells have global DNA hypermethylation [15, 16]. In contrast, less abundant hypomethylation in cisplatin‐resistant cells was associated with H3K27me3 and polycomb target gene loci as part of a bidirectional shift between gene promoter and gene body DNA methylation that was associated with upregulation of polycomb targets and downregulation of tumor suppressor genes [16]. Our data thus suggest a complex relationship exists between DNA methylation and H3K27me3, the remodeling of which may have been selected for during acquired cisplatin and 5‐aza resistance. The interconnection between polycomb and DNA methylation has been studied in other cell contexts including pluripotent embryonal stem cells [29, 30, 31, 32]. It would be interesting to directly test this relationship in future work with H3K27me3 and polycomb cistrome and chromatin accessibility ATAC‐seq analyses in TGCT cells. Further, important future work will be to confirm whether the epigenetic and gene expression changes highlighted here extend to clinical cisplatin‐resistant TGCTs.

Recent studies suggest that global DNA hypermethylation may be a key factor in mediating cisplatin resistance in TGCTs [16, 17]. The current work reveals that DNMT3B is also reciprocally altered with 5‐aza and cisplatin resistance. Prior studies have demonstrated that knockdown of highly expressed DNMT3B in TGCT cells confers resistance to hypomethylating agents [5, 6, 7, 8]. Interestingly, the 5‐aza‐resistant cells derived in the present study spontaneously downregulated DNMT3B, while cisplatin‐resistant cells greatly induced DNMT3B expression. This again suggests an interconnected epigenetic mechanism linking 5‐aza and cisplatin sensitivity/resistance in TGCTs involving polycomb and DNA methylation. Finally, DNMT3B knockdown alone phenocopied 5‐aza resistance including repression of polycomb target genes, 5‐aza resistance, and cisplatin sensitization, suggesting that DNMT3B plays an important role in the crosstalk between DNA methylation and polycomb in TGCT cells. We found that DNMT3B knockdown induced the protein expression of EZH2 and BMI1; however, only BMI1 mRNA was increased, suggesting that DNMT3B knockdown may also have post‐transcriptional effects on polycomb components. Interestingly, DNMT3B knockdown in parental TGCT cells resulted in many more downregulated genes than upregulated genes, suggesting that the high levels of DNMT3B in TGCT cells may have functions beyond the canonical role of promoting repression of gene expression [23].

Interestingly, the H3K27me3 demethylase KDM6B was upregulated in cisplatin‐resistant cells. Targeting H3K27me3 demethylases like KDM6B could be a strategy for treating cisplatin‐resistant tumors. We have previously demonstrated that inducing H3K27 methylation with GSK‐J4, an inhibitor of both KDM6A and KDM6B H3K27me3 demethylases, increased cisplatin sensitivity of cisplatin‐resistant TGCT cells [15]. Upregulation of KDM6B also provides a mechanistic explanation for the low basal levels of H3K27me3 in cisplatin‐resistant 2102EP cells. It will be important to validate our findings in cisplatin‐sensitive versus cisplatin‐resistant nonseminoma patients. Patient material could be interrogated for H3K27me3 levels and BMI1, EZH2, KDM6B, and DNMT3B levels by immunohistochemistry. We predict that patients resistant to cisplatin may have high levels of DNMT3B and KDM6B, and low levels of H3K27me3, EZH2, and BMI1. These patients would be predicted to be hypersensitive to 5‐aza and thus candidates for hypomethylation therapy with or without cisplatin. These patents would also be candidates for combination therapy with a KDM6A/KDM6B inhibitor and cisplatin.

The precise nature of how H3K27me3 and DNA methylation alters cisplatin and 5‐aza sensitivity will require further studies. Interestingly, DNMT3B knockdown alone was able to repress many of the same polycomb target genes that were repressed in 5‐aza‐resistant cells and upregulated in cisplatin‐resistant cells. These data suggest that alterations in chromatin context as revealed by polycomb target gene expression and likely downstream of DNMT3B/DNA methylation are a driving factor in TGCT cell response to cisplatin and 5‐aza. Whether these results hold true for other cancer therapeutics in TGCT cells awaits further study. In other cell contexts, studies have shown that H3K27me3 and other histone marks have the ability to recruit or regulate distinct components of the DNA damage and repair pathways [33, 34, 35, 36, 37, 38, 39, 40]. It will be interesting to assess how altering DNMT3B and polycomb components effect these endpoints in TGCT cells in future studies. Another potential mechanism to consider is the direct regulation of DNA damage response and repair gene expression by DNA methylation and polycomb remodeling in TGCT cells as recent reports have shown some genes involved in these pathways can be regulated by DNA methylation in TGCT cells and tumors [41, 42, 43]. However, we did not detect a clear signal for these family of genes in our transcriptome analyses.

Our ongoing work is centered on further elucidating the mechanism by which DNA and histone methylation are influencing 5‐aza and cisplatin sensitivity. However, these mechanisms are likely complex and are beyond to scope of the current study. Our two leading hypothesis currently are that these epigenetic marks may be directly influencing the recruitment of DNA repair or DNA damage pathway components, or alternatively, a comparable amount of DNA damage is occurring but cells are responding differently based on the chromatin context, for example, more open chromatin allowing for more binding of p53 to target gene promoters. Thus far in appears that there is a comparable amount of DNA damage and repair in parental, 5‐aza‐, and cisplatin‐resistant cells basally and in response to cisplatin and 5‐aza.

## 5 Conclusions

In summary, we have shown that 5‐aza‐ and cisplatin‐resistant TGCT cells reciprocally regulate H3K27me3 and DNMT3B and that this results in reciprocal sensitivity to 5‐aza and cisplatin. In that, 5‐aza‐resistant cells are more sensitive to cisplatin and cisplatin‐resistant cells are more sensitive to 5‐aza. Together, our findings support the involvement of a novel crosstalk mechanism between two epigenetic pathways, global DNA methylation, and polycomb, in mediating the unique hypersensitivity of TGCTs to cisplatin and hypomethylating agents. This is precisely in line with the growing realization that the etiology and progression of TGCTs may be especially dominated by epigenetic dysregulation. The cell models reported here may have value in identifying biomarkers to guide the use of cisplatin and hypomethylating agents for the treatment of TGCT patients.

## Conflict of interest

The authors declare no conflict of interest.

## Author contributions

RS, ZF, EB, SJF, and MJS performed the conceptualization; RS, ZF, EB, CY, HB, DS, AKC, KS, and AH performed the methodology; RS, ZF, AKC, EB, and CY performed the validation; RS, ZF, EB, CY, and MJS involved in formal analysis; RS, ZF, EB, CY, HB, DS, AKC, KS, and AH performed the investigation; RS and ZF performed data curation; RS, ZF, and MJS wrote the original draft preparation; SJF and MJS reviewed and edited the manuscript; RS, ZF, and EB performed visualization; RS, ZF, SJF, EB, and MJS involved in supervision; SJF and MJS involved in project administration; SJF and MJS contributed to funding acquisition.

## Supporting information


**Table S1.** RT‐PCR primers used in this study.

**Table S2.** Differentially expressed genes in 5‐aza resistant cells with FDR ≤ 0.01 and absolute fold change ≥ 1.5.

**Table S3.** Top 50 positively and negatively enriched gene sets in 5‐aza resistant cells compared to parental cells by GSEA.

**Table S4.** Top 20 predicted transcription factors binding to promoters of downregulated genes in 5‐aza resistant cells.

**Table S5.** Differentially expressed genes in 2102EP‐DNMT3BKD cells compared to shRNA control cells with FDR ≤ 0.01 and absolute fold change ≥ 1.5.

**Table S6.** Top 50 positively and negatively enriched gene sets in 2102EP‐DNMT3BKD cells compared to shRNA control cells by GSEA.

**Table S7.** Top 20 predicted transcription factors binding to promoters of downregulated genes in 2102EP‐DNMT3BKD cells.

**Table S8.** Differentially expressed genes common to 5‐aza resistant down/cisplatin resistant up, 5‐aza resistant down/DNMT3BKD down, or cisplatin resistant up/DNMT3BKD down with ≥ 1.5 fold‐change and FDR ≤ 0.01.

**Table S9.** Overlap analysis results between genes common to 5‐aza resistant down/cisplatin resistant up, 5‐aza resistant down/DNMT3BKD down, or cisplatin resistant up/DNMT3BKD down versus the MSigDB C2 collection of curated gene sets. Top 20 based on p value are presented.

## Data Availability

The RNA‐seq datasets for the current study have been submitted to the NCBI Database of GEO Datasets under the accession numbers GSE172266 and GSE172458.
